# Alzheimer’s disease multiomic signatures mediated by Cholesterol Ester Transfer Protein

**DOI:** 10.1002/alz.090912

**Published:** 2025-01-03

**Authors:** Tamar R Abel, Ravi S Pandey, Catrina Spruce, Tetiana Poliakova, Cheryl L Wellington, Gregory W Carter

**Affiliations:** ^1^ The Jackson Laboratory, Bar Harbor, ME USA; ^2^ The Jackson Laboratory for Genomic Medicine, Farmington, CT USA; ^3^ The University of British Columbia, Vancouver, BC Canada; ^4^ University of British Columbia, Vancouver, BC Canada; ^5^ University of Maine, Orono, ME USA; ^6^ Tufts University, Boston, MA USA; ^7^ Tufts University Graduate School of Biomedical Sciences, Boston, MA USA

## Abstract

**Background:**

Altered lipid profiles and lipid processing genes are associated with Alzheimer’s disease (AD). There is a reported genetic interaction between the AD risk gene *APOE* and cholesterol ester transfer protein (*CETP*). Mice lack functional *CETP* which is critical to the balance of circulating lipoproteins; this imparts cardioprotective effects and may make mice resistant to AD. Additionally, previous studies in transgenic humanized CETP (h*CETP*) mice have demonstrated that *CETP* addition in mice increases expression of AD risk genes and markers of AD pathogenicity. Together, this suggests that *CETP* may modify AD risk in an *APOE4*‐dependent manner. This project aims to determine molecular pathways and signatures in AD mediated by *CETP* with an emphasis on molecular pathways shared with *APOE* as a potential disease‐altering mechanism.

**Methods:**

To identify pathways shared by *CETP* and *APOE* we created molecular networks using publicly available online tools including the AD Atlas and STRING (v12.0). The Heart and Diabetes Institute Metabolomics Laboratory Portal was leveraged to perform Genome Wide Association Study (GWAS) analysis to establish changes in lipid species abundance shared by *CETP* and *APOE* genetic variants. Transcriptional signatures in several mouse models were compared to human AD gene modules to determine pathological relevance of h*CETP* and *APOE4* mouse models.

**Results:**

Molecular networks implicated several genes as being co‐expressed suggesting they may co‐function with *CETP* and *APOE*. These co‐expression edges included *C4A/B* compliment genes, *PSMB8*, *APOJ/CLU*, and *APP*. GWAS analysis resulted in several notable findings including that *CETP* variants have a strong effect on the relative abundance of phosphatidylcholine (PC) lipid species. Additionally, while *APOE4* is positively associated with total PC, the common loss‐of‐function variant *CETP* I405V (rs5882) is associated with reduced PC levels. Transcriptional changes in lipid‐related transgenic and MODEL‐AD knock‐in mouse models exhibited significant associations with immune processes.

**Conclusions:**

These findings indicate that both *CETP* and *APOE* influence total PC and are associated with molecular pathways of immune activation and amyloid biology. Overall this evidence suggests that *CETP* and *APOE* act on shared pathways, likely mediated by lipid metabolism and peripheral immunity, that contribute to AD susceptibility.

